# The significance of direct transportation to a trauma center on survival for severe traumatic brain injury

**DOI:** 10.1007/s00068-022-01885-3

**Published:** 2022-02-28

**Authors:** Dhanisha Jayesh Trivedi, Gary Alan Bass, Maximilian Peter Forssten, Kai-Michael Scheufler, Magnus Olivecrona, Yang Cao, Rebecka Ahl Hulme, Shahin Mohseni

**Affiliations:** 1 Division of Trauma and Emergency Surgery, Department of Surgery, Orebro University Hospital, 701 85 Örebro, Sweden; 2 School of Medical Sciences, Orebro University, 702 81 Örebro, Sweden; 3 Division of Traumatology, Surgical Critical Care and Emergency Surgery, University of Pennsylvania, Penn Presbyterian Medical Center, Philadelphia, PA 19104 USA; 4 Division of Neurosurgery, Department of Neurosurgery, Orebro University Hospital, Örebro, Sweden; 5 Clinical Epidemiology and Biostatistics, School of Medical Sciences, Orebro University, 701 82 Örebro, Sweden; 6 Department of Surgery, Karolinska University Hospital, 171 76 Stockholm, Sweden; 7 Medical School, Heinrich-Heine-University, Düsseldorf, Germany

**Keywords:** Severe traumatic brain injury, Trauma center, Triage, Mortality

## Abstract

**Introduction:**

While timely specialized care can contribute to improved outcomes following traumatic brain injury (TBI), this condition remains the most common cause of post-injury death worldwide. The purpose of this study was to investigate the difference in mortality between regional trauma centers in Sweden (which provide neurosurgical services round the clock) and non-trauma centers, hypothesizing that 1-day and 30-day mortality will be lower at regional trauma centers.

**Patients and methods:**

This retrospective cohort study used data extracted from the Swedish national trauma registry and included adults admitted with severe TBI between January 2014 and December 2018. The cohort was divided into two subgroups based on whether they were treated at a trauma center or non-trauma center. Severe TBI was defined as a head injury with an AIS score of 3 or higher. Poisson regression analyses with both univariate and multivariate models were performed to determine the difference in mortality risk [Incidence Rate Ratio (IRR)] between the subgroups. As a sensitivity analysis, the inverse probability of treatment weighting (IPTW) method was used to adjust for the effects of confounding.

**Results:**

A total of 3039 patients were included. Patients admitted to a trauma center had a lower crude 30-day mortality rate (21.7 vs. 26.4% days, *p* = 0.006). After adjusting for confounding variables, patients treated at regional trauma center had a 28% [adj. IRR (95% CI): 0.72 (0.55–0.94), *p* = 0.015] decreased risk of 1-day mortality and an 18% [adj. IRR (95% CI): 0.82 (0.69–0.98)] reduction in 30-day mortality, compared to patients treated at a non-trauma center. After adjusting for covariates in the Poisson regression analysis performed after IPTW, admission and treatment at a trauma center were associated with a 27% and 17% reduction in 1-day and 30-day mortality, respectively.

**Conclusion:**

For patients suffering a severe TBI, treatment at a regional trauma center confers a statistically significant 1-day and 30-day survival advantage over treatment at a non-trauma center.

## Introduction

Traumatic brain injury (TBI) is a common trauma-related injury worldwide; it affects 69 million people per year and is the most common cause of death in trauma patients [1, 2]. While primary prevention measures are the cornerstone to decreasing the incidence of such events, secondary prevention and optimization in the care of TBI patients can further decrease mortality and morbidity, and in some cases, even restore function [3].

Previous Swedish epidemiological studies have shown a general increase in hospital admissions following severe intracranial injuries after trauma [4]. Patients with TBI often require highly specialized care including neurosurgical intervention, neurointensive care, and neurorehabilitation. In Sweden, specialized TBI care is only provided at university hospitals, which are responsible for tertiary regional trauma care. Today, all seven university hospitals in Sweden qualify as regional trauma centers, where comprehensive round-the-clock neurosurgical care is provided. Traumatic brain-injured patients represent a group that benefits from early specialized care for better outcomes [5, 6]. This emphasizes the importance of early patient allocation to specialized care, preferably from the trauma site [7–9]. Determining patient allocation for TBI care is heavily influenced by the injury severity and the geographical proximity to the nearest hospital [10]. Therefore, hospital capabilities are not always the primary determinant of where the patient is transferred from the trauma site.

Despite the known fact that dedicated trauma centers have a better overall outcome in patients with severe injuries, there are still some controversies regarding direct transport of trauma patients from the scene of the accident to these centers in Sweden. While all aspects of neurosurgical care are provided exclusively at trauma centers, some still argue in favor of transport to the nearest hospital, regardless of their trauma level status, citing the faster transport time as an advantage.

Treating trauma-specific injuries at dedicated regional trauma centers in Sweden has been associated with reduced mortality by up to 32% [7–9]. However, little is known about the potential differences in mortality among trauma patients suffering from a severe traumatic brain injury between regional trauma centers (university hospitals), all of which have neurosurgical services, and non-trauma centers (non-university hospitals) in Sweden. The aim of the study is to examine the 1-day and 30-day mortality of patients with severe TBI (sTBI) treated at regional trauma centers versus those treated at non-trauma centers in Sweden, with the null-hypothesis that there is no difference in 1-day and 30-day mortality between patients treated for sTBI in trauma centers and non-trauma centers.

## Methods

Data were retrieved from SweTrau, the Swedish trauma register using ICD-10 codes for all patients with an intracranial injury. SweTrau is the only national trauma database of Sweden, which receives data on a voluntary basis from 43 of the 50 trauma-receiving hospitals in Sweden as of 2018 [11]. Data registering in SweTrau follows the “The Revised Utstein Template for Uniform Reporting of Data following Major Trauma, 2009” which is a uniform set of variables used in Europe to compare trauma and outcomes from trauma. [12].

All adult patients (18 years or older) in Sweden admitted with a sTBI between January 2014 and December 2018 were included in the study. A sTBI was defined as a head injury due to an intracranial injury with an abbreviated injury score (AIS) of three or higher [13]. Patients were excluded if the admitting hospital was unknown/missing, if they were transferred to a different hospital, or if they had an AIS of six in any body region. Retrieved data included patient demographics, initial Glasgow Coma Scale (GCS) in the emergency room (ER), American Society of Anesthesiologists (ASA) classification, AIS for each body region, types of brain injury (cerebral contusions, epidural hematoma, traumatic subdural hematoma, traumatic subarachnoid hemorrhage, or diffuse axonal injury), length of stay, and 1-day as well as 30-day mortality. The primary outcome was 30-day mortality from the time of trauma, while 1-day mortality was a secondary outcome of interest.

### Statistical analysis

Patients were divided into two cohorts based on whether they were admitted to a non-trauma center or a regional trauma center. A total of 42 hospitals were recorded in the database, of which seven were regional trauma centers.

The Mann–Whitney *U* test was used to compare continuous variables as they were all non-normally distributed. The statistical significance of differences between categorical variables was determined using the Chi-squared test or Fisher’s exact test. To calculate the incidence rate ratios (IRRs) and 95% confidence intervals (CI) for 1-day and 30-day mortality, Poisson regression analysis was performed with both univariable and multivariable models. In all analyses, mortality was the response variable, and the admitting hospital (non-trauma center or regional trauma center) was the predictor. In the multivariable analysis, the model was adjusted for age, sex, ASA classification, initial GCS in the ER, type of intracranial injury, as well as the highest AIS in each body region (head, face, neck, thorax, abdomen, spine, upper extremity, lower extremity, and external/other).

Multiple imputation by chained equations was used to compensate for missing values and was performed using proportional odds models. Statistical significance was defined as a two-sided *p* value less than 0.05.

As a sensitivity analysis, the inverse probability of treatment weighting (IPTW) method was used to adjust for the effects of confounding. The propensity score for each patient was determined using a logistic regression model, which included admitting hospital (non-trauma center or regional trauma center) as the response variable, and age, sex, ASA classification, initial GCS in the ER, type of intracranial injury, as well as the highest AIS in each body region as the predictors. The weights were calculated as $\frac{{1}}{{\text{probabilty of admission to a regional trauma center}}}$ for patients admitted to regional trauma centers and $\frac{1}{{1 - {\text{ probabilty of admission to a regional trauma center}}}}$ for patients admitted to non-trauma centers. Differences between the cohorts after weighting were evaluated using absolute standardized differences (ASD). An ASD < 0.1 was considered balanced. Finally, Poisson regression models were fitted to the weighted cohorts, any unbalanced covariates were adjusted for in these models.

Analyses were performed using the statistical programming language R (R Foundation for Statistical Computing, Vienna, Austria) [14]. Ethical approval for this study was received from the Swedish Ethical Review Authority (reference number 2021-00694). The study complies with the STROBE guidelines and the Declaration of Helsinki. [15].

## Results

A total of 3039 patients were included for analysis. Patients admitted to a trauma center were younger (58 vs. 69 years old, *p* < 0.001], more often male (70.7 vs. 66.3%, *p* = 0.017), had more severe intracranial injuries (AIS 5: 25.1% vs. 19.6%, *p* < 0.001), and were subjected to neurosurgical intervention to a significantly higher degree (8.0% vs. 1.0%, *p* < 0.001) (Table 1). Patients admitted to a trauma center were also more fit for surgery according to their ASA classification (ASA ≥ 3: 24.8 vs. 28.5%, *p* < 0.001) while having more severe injuries in almost all extracranial regions (Table 1). Hospital length of stay was longer for patients treated in a trauma center (6 vs. 5 days, *p* < 0.001). Patients admitted to a trauma center also had a lower crude 30-day mortality rate (21.7 vs. 26.4% days, *p* = 0.006) (Table 2).

**Table 1:** Patient demographics and clinical features after a sTBI

	Non-trauma centers (*N* = 928)	Regional trauma centers (*N* = 2111)	*p* value
Age, median [IQR]	69 [49–82]	58 [38–74]	< 0.001
Sex, *n* (%)			
Female	313 (33.7)	619 (29.3)	0.017
Male	615 (66.3)	1492 (70.7)	
ASA classification, *n* (%)			
1	330 (35.6)	924 (43.8)	< 0.001
2	305 (32.9)	597 (28.3)	
3	249 (26.8)	484 (22.9)	
4	16 (1.7)	40 (1.9)	
5	0 (0.0)	1 (0.0)	
Missing	28 (3.0)	65 (3.1)	
Highest level of pre-hospital care provided, *n* (%)			
No pre-hospital care required	36 (3.9)	61 (2.9)	< 0.001
Basic pre-hospital care	231 (24.9)	23 (1.1)	
Nurse anesthetist	610 (65.7)	1,687 (79.9)	
Medical doctor	24 (2.6)	251 (11.9)	
Other	1 (0.1)	0 (0.0)	
Missing	26 (2.8)	89 (4.2)	
Time from the trauma site to admission in minutes, median [IQR]	38 [27–54]	37 [27–48]	0.011
Missing, *n* (%)	63 (6.8)	159 (7.5)	
Time from hospital admission to first emergency intervention in minutes, median [IQR]	80 [17–240]	100 [49–180]	0.333
Missing, *n* (%)	840 (90.5)	1510 (71.5)	
Initial GCS in the ER, *n* (%)			
Mild (GCS 14–15)	484 (52.2)	990 (46.9)	0.014
Moderate (GCS 9–13)	156 (16.8)	396 (18.8)	
Severe (GCS 3–8)	145 (15.6)	393 (18.6)	
Missing	143 (15.4)	332 (15.7)	
Type of trauma, *n* (%)			
Blunt	896 (96.6)	2,022 (95.8)	0.002
Penetrating	17 (1.8)	87 (4.1)	
Missing	15 (1.6)	2 (0.1)	
Type of intracranial injury, *n* (%)			
Cerebral contusion	279 (30.1)	1019 (48.3)	< 0.001
Epidural hematoma	43 (4.6)	187 (8.9)	< 0.001
Subdural hematoma	552 (59.5)	1211 (57.4)	0.294
Subarachnoid hemorrhage	325 (35.0)	1019 (48.3)	< 0.001
Diffuse axonal injury	13 (1.4)	86 (4.1)	< 0.001
Other intracranial injury	6 (0.6)	10 (0.5)	0.589
Injury severity score, median [IQR]	16 [10–25]	18 [13–26]	< 0.001
Head, *n* (%)			
3	557 (60.0)	1207 (57.2)	0.001
4	189 (20.4)	374 (17.7)	
5	182 (19.6)	530 (25.1)	
Face, *n* (%)			
Injury not present	674 (72.6)	1088 (51.5)	< 0.001
1	138 (14.9)	476 (22.5)	
2	110 (11.9)	474 (22.5)	
3	6 (0.6)	66 (3.1)	
4	0 (0.0)	7 (0.3)	
Neck, *n* (%)			
Injury not present	917 (98.8)	2011 (95.3)	< 0.001
1	7 (0.8)	52 (2.5)	
2	2 (0.2)	18 (0.9)	
3	1 (0.1)	16 (0.8)	
4	0 (0.0)	11 (0.5)	
5	1 (0.1)	3 (0.1)	
Thorax, *n* (%)			
Injury not present	728 (78.4)	1422 (67.4)	< 0.001
1	31 (3.3)	98 (4.6)	
2	69 (7.4)	168 (8.0)	
3	69 (7.4)	294 (13.9)	
4	25 (2.7)	90 (4.3)	
5	6 (0.6)	39 (1.8)	
Abdomen, *n* (%)			
Injury not present	893 (96.2)	1907 (90.3)	< 0.001
1	6 (0.6)	60 (2.8)	
2	15 (1.6)	59 (2.8)	
3	7 (0.8)	39 (1.8)	
4	6 (0.6)	38 (1.8)	
5	1 (0.1)	8 (0.4)	
Spine, *n* (%)			
Injury not present	804 (86.6)	1676 (79.4)	< 0.001
1	4 (0.4)	14 (0.7)	
2	105 (11.3)	335 (15.9)	
3	13 (1.4)	51 (2.4)	
4	0 (0.0)	16 (0.8)	
5	2 (0.2)	19 (0.9)	
Upper extremity, *n* (%)			
Injury not present	727 (78.3)	1332 (63.1)	< 0.001
1	67 (7.2)	410 (19.4)	
2	133 (14.3)	343 (16.2)	
3	1 (0.1)	26 (1.2)	
Lower extremity, *n* (%)			
Injury not present	794 (85.6)	1448 (68.6)	< 0.001
1	53 (5.7)	377 (17.9)	
2	44 (4.7)	126 (6.0)	
3	34 (3.7)	100 (4.7)	
4	3 (0.3)	34 (1.6)	
5	0 (0.0)	26 (1.2)	
External, *n* (%)			
Injury not present	672 (72.4)	1843 (87.3)	< 0.001
1	256 (27.6)	268 (12.7)	
Neurosurgical intervention, *n* (%)	9 (1.0)	169 (8.0)	< 0.001
Missing	13 (1.4)	7 (0.3)	
Thoracotomy, *n* (%)	1 (0.1)	27 (1.3)	< 0.001
Missing	13 (1.4)	7 (0.3)	
Laparotomy, *n* (%)	9 (1.0)	27 (1.3)	0.586
Missing	13 (1.4)	7 (0.3)	

*sTBI* severe traumatic brain injury,* ASA* American Society of Anesthesiologists,* GCS* Glasgow Coma Scale,* ER* emergency room

**Table 2:** Crude patient outcomes after a sTBI

	Non-trauma centers (*N* = 928)	Regional trauma centers (*N* = 2111)	*p* value
Length of stay, median (IQR)	5.0 [3.0–9.0]	6.0 [3.0–13]	< 0.001
Missing, *n* (%)	4 (0.4)	0 (0.0)	
1-day mortality, *n* (%)	110 (11.9)	229 (10.8)	0.454
30-day mortality, *n* (%)	245 (26.4)	459 (21.7)	0.006

*sTBI* severe traumatic brain injury,* IQR* interquartile range

In the multivariable regression analysis, trauma center status was associated with a lower rate of 1-day mortality among sTBI patients. After adjusting for potential confounders, the mortality risk was reduced by 28% among patients admitted directly from the site of injury to trauma centers [IRR (95% CI) 0.72 (0.55–0.94), *p* = 0.015], compared to patients who were treated at a non-trauma center (Table 3).

**Table 3:** IRRs for mortality after a sTBI

	Unadjusted IRR (95% CI)	*p* value	Adjusted IRR (95% CI)	*p* value
1-day mortality				
Hospital academic status				
Non-trauma centers	Ref		Ref	
Regional trauma centers	0.91 (0.72–1.15)	0.444	0.72 (0.55–0.94)	0.015
30-day mortality				
Hospital academic status				
Non-trauma centers	Ref		Ref	
Regional trauma centers	0.81 (0.69–0.94)	0.007	0.82 (0.69–0.98)	0.028

All analyses used Poisson regression models with robust standard errors of variance. Missing values were managed using multiple imputations by chained equations. The adjusted model was adjusted for age, sex, American Society of Anesthesiologists classification, initial GCS in the ER, the presence of cerebral contusions, an epidural hematoma, a traumatic subdural hematoma, a traumatic subarachnoid hemorrhage, or diffuse axonal injury, as well as the highest abbreviated injury score in each region

*IRR* Incident Rate Ratio, *sTBI* Severe Traumatic Brain Injury,* CI* Confidence Interval

These same trends were evident in both the univariable and multivariable regression analyses with 30-day mortality as the outcome. After adjusting for confounders, the risk of mortality within 30-day was still reduced by 18% among patients admitted directly from the site of injury to trauma centers [IRR (95% CI) 0.82 (0.69–0.98), *P* = 0.028], compared to patients who were treated at a non-trauma center (Table 3).

All variables were balanced after IPTW, except for the AIS in the neck, abdomen, spine, as well as lower and upper extremity (Supplementary Table 1). However, after adjusting for these covariates in the Poisson regression analysis performed after IPTW, admission and treatment at a trauma center was still associated with a 27% and 17% reduction in 1-day and 30-day mortality, respectively (Supplementary Table 2).

## Discussion

The current study demonstrates a statistically significant reduction in 1-day and 30-day mortality in sTBI patients admitted directly to a regional trauma center compared to non-trauma centers. These findings are consistent with, and expand on, previous Swedish and international studies, which have associated traumatic injury treatment at dedicated regional trauma centers with reduced mortality [7–9, 16]. Injury type-specific studies, such as this one, are not unprecedented. Recent work identified reduced mortality for patients with unstable pelvic and severe acetabular fractures following trauma when cared for in a trauma center compared to a non-trauma center [17]. By isolating the traumatic injury type, one can further evaluate the injury-specific benefits of transportation directly to trauma centers from the site of injury versus treatment at the nearest located hospital.

When it comes to sTBI in Sweden, several factors may explain the lower mortality detected in patients admitted directly to trauma centers. Prompt neurosurgical consultation and transport to a fully equipped operating theatre are positively affected by the proximity of a neurosurgical team in trauma centers, that can even be available for discussion right in the emergency room while primary survey and resuscitation are underway. Thus, one critical advantage that trauma centers have over non-trauma centers is the much shorter delay to neurosurgical intervention. Minimized time to surgery has been strongly associated with lower mortality and better functional outcome following sTBI [18, 19]. Especially, early surgical intervention has been beneficial in the management of severe epidural and subdural hematomas [20].

While neurosurgical procedures are performed exclusively in regional trauma centers of Sweden, some non-trauma centers in remote areas do have access to surgeons who have received training in dealing with a limited scope of life-threatening hemorrhages such as epidural hematomas, only an average of 1.8 neurosurgical interventions are performed each year in a non-trauma center in all of Sweden, hence this training is exceedingly rare. This is mirrored in our dataset which shows a total of 9 procedures were performed in non-trauma centers (Table 1).

A previous study found higher mortality during the first hour following sTBI (defined as an intracranial AIS of at least four), described as a high first-hour mortality peak, while no mortality peak was demonstrated in milder TBI (AIS less than four). The authors concluded that early surgical intervention has a substantial benefit in preventing early death in sTBI [18]. Although the current study included patients with multiple injuries, more recent studies confirm these findings and further describe a significant reduction in mortality with sTBI associated with a shorter time to surgery, even recommending a consultation with a neurosurgical specialist within 30 min of patient arrival. [18].

Other factors which decrease the time to surgery and contributes to better outcomes following sTBI include early identification of patients with a high risk of TBI, rapid diagnosis of type and severity of the injury through CT, timely neurosurgical consultation, and other non-neurosurgical interventions such as securing airway and controlling the source(s) of bleeding. To achieve these goals, dedicated trauma teams that care for a higher volume of severely injured patients is of paramount importance. Further, a high level of care is beneficial in sTBI patients regardless of the management, including both those subjected to an operation and also those injuries that are managed non-operatively. [20–24].

Non-operative treatments of sTBI, as recommended by the Brain trauma foundation TBI guidelines, are widely accepted treatments for certain subgroups of sTBI [21]. However these patients are in need of close observation, monitoring and other procedures. These include procedures such as intracranial pressure monitoring, cerebrospinal fluid drainage, microdialysis, and other interventions aimed at TBI-specific neurosurgical monitoring or dealing with certain complications. All of these require specialized care and close monitoring which is only provided at regional trauma centers (Swedish university hospitals) [22, 24, 25].

The patient volume-outcome relationship may further explain the lower mortality associated with treatment at trauma centers in Sweden. Several studies have previously associated lower in-hospital mortality with higher patient volumes [25–28]. Volume-outcome relationships have led to the regionalization of highly specialized care, especially in cancer care and surgery, as well as cardiac catheterization [8, 11, 28]. The volume-outcome relationship has also been evaluated within the field of trauma and neurosurgery with the same positive results on mortality [26–28]. This is reflected in the current dataset where the median [IQR] number of sTBI patients admitted per year to non-trauma centers was 8.2 [2.4–13.35] while the same number for regional trauma centers was 50.9 [28.5–82]. Thus, confirming that regional trauma centers have a higher volume of sTBI patients.

There are several limitations in the current study that need to be addressed. First, this retrospective study was conducted using data procured from the Swedish national trauma registry, SweTrau. Registration in SweTrau is voluntary and missing data is therefore unavoidable. Of the 50 Swedish hospitals which accept trauma patients, 46 are coupled to SweTrau and four do not provide data to the registry at all [11]. Second, the specific cause of death were not available for analysis, therefore, the possibility that deaths occurred unrelated to the sTBI or its sequelae remain unknown. There is also a risk of selection bias as randomized allocation to hospitals is essentially non-existent in practice, and in this retrospective setting, controlling for the main reason for transport is not feasible. Nevertheless, the higher number of neurosurgical interventions would suggest that the need for surgery could be one important factor. Finally, matching the two cohorts using the patients’ propensity scores would have been ideal to control for residual confounding; however, as a result of the limited sample size this would have resulted in a type II error. Consequently, the propensity scores were used for IPTW instead.

Field triage in Sweden ensures that patients deemed more severely injured by triage and field criteria are transported to trauma centers with a higher level of subspecialized care, whenever possible. This is exemplified in our data as well (Table 1), where a higher number of patients with a more severe head injury (represented by a lower GCS on admission), more severe injury in other body regions (represented by a higher AIS score), and a higher number of thoracotomies and laparotomies were seen in those admitted to trauma centers. There are many reasons for this, including the need for intubation and stabilization of vital signs, or the physical distance to a trauma center. The latter has been addressed in the literature, and a longer transport time has not been associated with increased mortality in trauma patients, except in those with circulatory shock symptoms requiring immediate source control and resuscitation. [7, 29, 30] This finding has been attributed to advancements in the on-route-resuscitation.

The level of pre-hospital care provided on-site is determined largely by injury mechanism and severity. On-route-resuscitation, including rapid sequence intubation, is usually performed by nurse anesthetists and medical doctors on site, who were available in 79.9% and 11.9% of cases admitted to regional trauma centers respectively, as compared to 66% and 2.6% respectively at non-trauma centers (Table 1), which is consistent with the findings that the more severely injured patients are admitted to a regional trauma center for further management. On the other hand, a higher level of pre-hospital care can improve overall outcome, by reducing early death and preventing secondary brain injury. One can argue that the patients admitted to regional trauma centers, received on average a higher level of pre-hospital care, since they are more severely injured, but only 14.9% of those patients admitted to a regional trauma center required pre-hospital airway management. It is not possible to say to which degree the pre-hospital care improved the overall outcome based on our current dataset.

Elderly patients constitute an important group which does not always respond well to, or survive, the aggressive interventions necessary to sustain life as is required in sTBI. While in other countries it has become common to have a written declaration intended to limit aggressive therapeutic options, this is not as prevalent in Sweden. According to Swedish law, during a situation where the patient is incapacitated and unable to give consent for therapy, the on-site emergency unit performs life-sustaining interventions, such as rapid sequence intubation and cardiopulmonary resuscitation until such a time as the patient’s injuries are more apparent, and a medical doctor/surgeon has been placed in charge of treatment. In most cases, this first occurs in the hospital emergency room. At this time the doctor in charge can determine the treatment limitations. As a result of this paradigm, the dataset did not record the presence of advanced directives limiting care.

The findings from this study mandate further work in the area to determine how to triage patients who would benefit from direct transport from the site of injury to a trauma center with neurosurgical capacity, and how to make such a system both sustainable and safe.

## Conclusion

In conclusion, regional trauma center status is associated with a better outcome in patients suffering from severe traumatic brain injury. This finding lends additional weight to implementing trauma systems in favor of direct transport of those suffering from severe injury, especially severe traumatic brain injury, to hospitals where a higher level of care can be provided in a timely matter.
